# The relationships of sex hormone‐binding globulin, total testosterone, androstenedione and free testosterone with metabolic and reproductive features of polycystic ovary syndrome

**DOI:** 10.1002/edm2.267

**Published:** 2021-05-24

**Authors:** Pomme I. H. G. Simons, Olivier Valkenburg, Judith A. P. Bons, Coen D. A. Stehouwer, Martijn C. G. J. Brouwers

**Affiliations:** ^1^ Division of Endocrinology and Metabolic Diseases Department of Internal Medicine Maastricht University Medical Centre Maastricht The Netherlands; ^2^ Laboratory for Metabolism and Vascular Medicine Maastricht University Maastricht The Netherlands; ^3^ CARIM School for Cardiovascular Diseases Maastricht University Maastricht The Netherlands; ^4^ Department of Reproductive Medicine Maastricht University Medical Centre Maastricht The Netherlands; ^5^ Central Diagnostic Laboratory Maastricht University Medical Centre Maastricht The Netherlands; ^6^ Division of General Internal Medicine Department of Internal Medicine Maastricht University Medical Centre Maastricht The Netherlands

**Keywords:** androgens, metabolic syndrome, polycystic ovary syndrome, sex hormone‐binding globulin

## Abstract

**Objective:**

A recent Mendelian randomization study has suggested a causal role for sex hormone‐binding globulin (SHBG), total testosterone and free testosterone in the pathogenesis of polycystic ovary syndrome (PCOS). The aim of this study was to assess the relationships of SHBG, androstenedione, total and free testosterone with the individual metabolic and reproductive features of PCOS.

**Design:**

Cross‐sectional data in PCOS patients (n=96) prospectively collected in a secondary/tertiary clinic for menstrual cycle disorders.

**Methods:**

Multivariable regression analyses were conducted to study the associations between SHBG, androstenedione, total and free testosterone with metabolic (BMI, waist circumference, systolic and diastolic blood pressure, total cholesterol, LDL cholesterol, HDL cholesterol, triglycerides and homeostatic model assessment for insulin resistance [HOMA2‐IR]) and reproductive features (menstrual cycle length, antral follicle count, anti‐Müllerian hormone, luteinizing hormone, follicle‐stimulating hormone and Ferriman‐Gallwey score) of PCOS.

**Results:**

Serum SHBG and free testosterone, but not total testosterone or androstenedione, were significantly associated with BMI, waist circumference, serum triglycerides, HDL cholesterol, LDL cholesterol and HOMA2‐IR. The strength of the associations with serum lipids was reduced after adjustment for BMI, but not for HOMA2‐IR. Total testosterone was significantly associated with antral follicle count. SHBG, total testosterone and androstenedione were significantly associated with serum AMH. Only the strength of the association for SHBG was reduced after adjustment for BMI.

**Conclusions:**

Serum SHBG is associated with primarily metabolic features, whereas total testosterone and androstenedione are associated with reproductive features of PCOS. These results suggest a differential underlying pathophysiology for the metabolic and reproductive features of PCOS.

## Graphical abstract

Sex hormone‐binding globulin (SHBG), total testosterone, and free testosterone have been found to play a causal role in the pathogenesis of polycystic ovary syndrome (PCOS), but it remains uncertain how these androgen markers associate with individual features of PCOS. In this cross‐sectional study in a cohort of patients with PCOS, serum SHBG was primarily associated with metabolic features of PCOS, whereas total testosterone and androstenedione were associated with reproductive features of PCOS. These results suggest a differential underlying pathophysiology for the metabolic and reproductive features of PCOS.

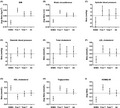

## 1 INTRODUCTION

Polycystic ovary syndrome (PCOS) is the most common endocrine disorder among premenopausal women, with an estimated prevalence of 10%.^1^ Many, but not all women with PCOS exhibit metabolic disturbances, including obesity, insulin resistance, hypertension and dyslipidaemia, and endocrine abnormalities such as an increased ratio of luteinizing hormone (LH) to follicle‐stimulating hormone (FSH), and increased anti‐Müllerian hormone (AMH) levels.^2^

Androgen excess is a fundamental, diagnostic feature of PCOS that is present in approximately half to three quarters of PCOS patients.^3^, ^4^ A recent Mendelian randomization study has shown that genetically predicted SHBG, total testosterone and free testosterone levels are associated with the risk of PCOS.^5^ This establishes a potential causal role of free testosterone, and its determinants, in the pathogenesis of PCOS. Nevertheless, there is an ongoing discussion on the role of free testosterone in the development of the individual features of PCOS.

The role of androgens in the pathophysiology of PCOS is likely a complex, multifactorial process, driven by genetics, hormonal imbalance and lifestyle factors.^5^, ^6^, ^7^ Some have speculated that free testosterone plays a central role in the pathogenesis of all PCOS features, by actively contributing to the arrest of follicular development, theca cell dysfunction, ovarian stromal hyperplasia, abnormal gonadotrophin‐releasing hormone (GnRH) secretion and insulin resistance.^2^, ^8^, ^9^, ^10^ Others have argued that hyperandrogenism is merely a consequence of metabolic dysfunction or ovarian and endocrine changes, and does not, in itself, contribute to the pathophysiology of PCOS.^11^, ^12^, ^13^, ^14^ SHBG may reflect primarily metabolic changes, while total testosterone and androstenedione may reflect reproductive dysfunction.^11^

The aim of the present study is, therefore, to study the associations of serum SHBG, androstenedione, total testosterone and free testosterone with the individual metabolic and reproductive features of PCOS.

## 2 MATERIALS AND METHODS

### 2.1 Study population

Data were prospectively collected at the outpatient clinic for menstrual cycle disorders of the Maastricht University Medical Centre (Maastricht, The Netherlands) between March 2017 and February 2020. PCOS was retrospectively diagnosed according to the Rotterdam criteria, which requires the presence of at least two of the following three characteristics: irregular menstrual cycle, hyperandrogenism and polycystic ovarian morphology (PCOM).^15^ Irregular menstrual cycle was defined as a menstrual cycle length ≥35 days. Hyperandrogenism was defined as a free androgen index (total testosterone (nmol/L) * 100 / SHBG (nmol/L)) >4.5,^16^ total testosterone >1.9 nmol/L, androstenedione >9.6 nmol/L, or a Ferriman‐Gallwey score ≥4 for women of Caucasian, Black or Mixed ethnicity, and ≥6 for women of Middle‐Eastern and Asian ethnicity.^17^, ^18^, ^19^ PCOM was defined as the presence of ≥20 follicles (2–9 mm in diameter) in either ovary or an ovarian volume ≥10 ml, according to the revised international evidence‐based PCOS guidelines (European Society of Human Reproduction and Embryology [ESHRE] guidelines, 2018).^18^ Women who were pregnant, used hormonal contraceptives at the time of the clinical assessment, had abnormal thyroid stimulating hormone (TSH) levels (Table 1), elevated prolactin levels (Table 1), or individuals diagnosed with non‐classic congenital adrenal hyperplasia, were excluded from the current study.

**TABLE 1:** General characteristics of the study population

	PCOS population (n = 96)	Reference interval^a^
Age (years)	28.4 ± 4.2	
Ethnicity, n (%)		
Caucasian	88 (92)	
Black	2 (2)	
Middle‐Eastern	3 (3)	
Asian	2 (2)	
Mixed	1 (1)	
Smoking (cigarettes/day)	0.0 (0.0–0.0)	
Alcohol (units/week)	0.0 (0.0–2.0)	
Fasting, yes (%)	89 (93)	
TSH (mU/L)	1.8 (1.5–2.4)	0.4–4.3
Prolactin (U/L)	0.23 ± 0.09	0.10–0.64^b^ 0.01–0.50^b^
Metabolic features		
BMI (kg/m^2^)	26.0 (22.3–33.1)	
Waist circumference (cm)	84.0 (75.0–101.0)	
Systolic blood pressure (mmHg)	122 ± 13	
Diastolic blood pressure (mmHg)	75 ± 10	
Glucose (mmol/L)	4.9 ± 0.5	3.1–6.1
Insulin (pmol/L)	39.8 (16.8–64.3)	12–150
HOMA2‐IR	0.7 (0.3–1.2)	
Triglycerides (mmol/L)	0.8 (0.6–1.2)	0.9–1.94
Total cholesterol (mmol/L)	4.6 ± 0.9	<5.0
HDL cholesterol (mmol/L)	1.6 ± 0.4	>0.9
LDL cholesterol (mmol/L)	2.9 ± 0.9	<2.5
Metabolic syndrome, yes (%)	15 (16)	
Reproductive features		
Average length of menstrual cycle (days)	51 (40–96)	
Regularity of menstrual cycle, n (%)		
Regular	3 (3)	
Oligomenorrhoe	72 (75)	
Amenorrhoe	19 (20)	
Metrorrhagia	2 (2)	
AMH (µg/L)	7.6 (4.8–11.1)	<6.9
Antral follicle count^c^	20.8 ± 7.8	
Ovarian volume^c^	8.0 (6.5–10.7)	
PCOM, yes (%)	73 (76)	
FSH (U/L)	5.6 ± 2.3	Follicular phase: 2.8–14.4 Ovulatory phase: 5.8–21.0 Luteal phase: 1.2–9.0
LH (U/L)	7.7 (5.0–11.4)	Follicular phase: 1.1–11.6 Ovulatory phase: 17.0–77.0 Luteal phase: <0.05–14.7
Total testosterone (nmol/L)	1.7 ± 0.7	0.3–1.9
SHBG (nmol/L)	42 (28–63)	40–120
Free testosterone (pmol/L)	21.1 (14.3–29.8)	3.5–24
Free androgen index	3.5 (2.4–6.0)	
Androstenedione (nmol/L)	11.8 ± 4.1	3.0–9.6
Hyperandrogenism, yes (%)^d^	77 (80)	
Ferriman‐Gallwey score	5 (1–9)	
Hirsutism, yes (%)	27 (28)	
Self‐reported acne, yes (%)	44 (46)	

Abbreviations: AMH, anti‐Müllerian Hormone; BMI, body mass index; FSH, follicle‐stimulating hormone; HDL, high‐density lipoprotein; HOMA2‐IR, homeostatic model assessment for insulin resistance; LDL, low‐density lipoprotein; LH, luteinizing hormone; PCOM, polycystic ovarian morphology; SHBG, sex hormone‐binding globulin; TSH, thyroid stimulating hormone.

^a^ Reference intervals according to the Central Diagnostic Laboratory at the Maastricht University Medical Centre (The Netherlands).

^b^ Reference intervals prior to November 2018 and after November 2018, respectively; see methods section.

^c^ Average of both ovaries.

^d^ Biochemical or clinical (according to the Ferriman‐Gallwey score) hyperandrogenism.

This study was approved by the Medical Ethics Committee of Maastricht University Medical Centre.

### 2.2 Clinical assessment

All patients filled out questionnaires regarding demographics (age and ethnicity), lifestyle (smoking status and alcohol consumption), self‐reported history of acne and hirsutism (defined according to the aforementioned Ferriman‐Gallwey score cut‐off values) and gynaecological history (length and regularity of menstrual cycle). A regular menstrual cycle was defined as a menstrual cycle <35 days, oligomenorrhea as a menstrual cycle ≥35 days, amenorrhea as no menstrual period during the prior six months, and metrorrhagia as vaginal bleeding at irregular intervals.

Physical examination was performed to determine body mass index (BMI; calculated as body weight [kilograms] divided by length [meters] squared), waist circumference (at the level of the umbilicus), and systolic and diastolic blood pressure measured in semi‐seated position after 10 minutes of rest with an Omron 705IT automated measuring device. A transvaginal ultrasound was performed to count the total number of antral follicles (2–9 mm in diameter) in each ovary and calculate the ovarian volume (as 0.523 * length * width * depth for each ovary^20^), which were subsequently expressed as the average of two ovaries. In four cases, an abdominal ultrasound was performed instead and, where possible, ovarian volume and antral follicle count were assessed.

Blood was drawn in the morning. Patients were asked to visit the outpatient clinic after an overnight fast. Laboratory analyses were performed by the Central Diagnostic Laboratory at the Maastricht University Medical Centre (The Netherlands). All reference intervals were locally established by the Central Diagnostic Laboratory. Total testosterone and TSH were measured with an electrochemiluminescence immunoassay (Cobas 8000 instrument, Roche Diagnostics, Mannheim, Germany); FSH, LH, SHBG and insulin with an chemiluminescent immunometric assay (Immulite XPi instrument, Siemens Healthcare Diagnostics, New Orleans, LA, USA); serum glucose with an enzymatic spectrophotometric assay (Cobas 8000 instrument, Roche Diagnostics, Mannheim, Germany); triglycerides, total cholesterol and HDL cholesterol with an enzymatic colorimetric assay (Cobas 8000 instrument, Roche Diagnostics, Mannheim, Germany); and androstenedione with a radio immunoassay (IBL International, Hamburg, Germany). Prolactin was measured with electrochemiluminescence immunoassay (Cobas 8000 instrument, Roche Diagnostics, Mannheim, Germany) until November 2018, and with immunoassay (AutoDelfia, Perkin Elmer, Turku, Finland) after this date. AMH was measured with an enzyme‐linked immunosorbent assay (Gen II, Beckman Coulter, Brea, CA, USA) until July 2019, and with a chemiluminescent immunometric assay (Lumipulse G1200, Fujirebio, Tokyo, Japan) after this date. AMH levels determined by the enzyme‐linked immunosorbent assay were multiplied with a correction factor of 0.88 to obtain chemiluminescent immunometric assay calibrated AMH values.^21^ Free testosterone was calculated using the Ross algorithm.^22^ LDL cholesterol was calculated using the Friedewald formula.^23^ The homeostasis model assessment 2 (HOMA2‐IR) was calculated as a measure of insulin resistance (available at http://www.dtu.ox.ac.uk/homacalculator/). The metabolic syndrome was defined as the presence of at least three of the following five characteristics: a waist circumference ≥88 cm, triglycerides ≥1.7 mmol/L, HDL cholesterol <1.3 mmol/L, blood pressure ≥130/≥85 mmHg and a fasting glucose ≥6.1 mmol/L.^24^

### 2.3 Statistical analyses

Continuous data are presented as mean ±standard deviation (SD) or as median (interquartile range [IQR]) in case of non‐normal distribution. Categorical data are presented as frequencies. Non‐normally distributed variables were log‐transformed before further analyses. Multivariable linear regression analyses were performed to study the associations of SHBG, androstenedione, total testosterone and free testosterone with metabolic (BMI, waist circumference, systolic and diastolic blood pressure, total cholesterol, LDL cholesterol, HDL cholesterol, triglycerides and HOMA2‐IR) and reproductive features (length of menstrual cycle, antral follicle count, AMH, LH, FSH and Ferriman‐Gallwey score) of PCOS, independent of potential confounders. Z‐scores (= individual value minus population mean, divided by population SD) were calculated for SHBG, androstenedione, total testosterone and free testosterone before entry into the model to allow comparison of the strength of association between these variables. Since not all patients visited the outpatient clinic in the fasting state, analyses for most metabolic characteristics were adjusted for fasting (yes/no). Additional adjustments were made for age, BMI and HOMA2‐IR, for those metabolic and reproductive features that showed a statistically significant association with any of the androgen markers. Sensitivity analyses were conducted in fasted individuals only. All results were considered statistically significant at *p* < .05. All statistical analyses were performed using IBM Statistical Package of Social Science (SPSS) version 25.0 for Windows (IBM Corp.).

## 3 RESULTS

### 3.1 Study population

Between March 2017 and February 2020, we retrospectively identified 111 women who fulfilled the diagnostic criteria for PCOS. Fifteen individuals were excluded because they were pregnant (n = 3), used hormonal contraceptives (n = 9) or had elevated prolactin levels (n = 3) at the time of the clinical assessment. The general characteristics of the patients with PCOS (n = 96) are presented in Table 1. On average, the study population was young (mean age: 28.4 ± 4.2 years) and overweight (median BMI: 26.0, IQR: 22.3–33.1 kg/m^2^). Due to the specialized outpatient setting, the majority of women experienced oligomenorrhea (75%) or amenorrhea (20%). Additionally, the majority of women (76%) were found to have PCOM on ultrasound examination. Finally, 28% of women suffered from hirsutism and 46% reported a history of acne. Only a small percentage of women (16%) met the criteria for the metabolic syndrome.

### 3.2 Associations of serum SHBG, androstenedione, total testosterone and free testosterone with metabolic features of PCOS

**FIGURE 1:**
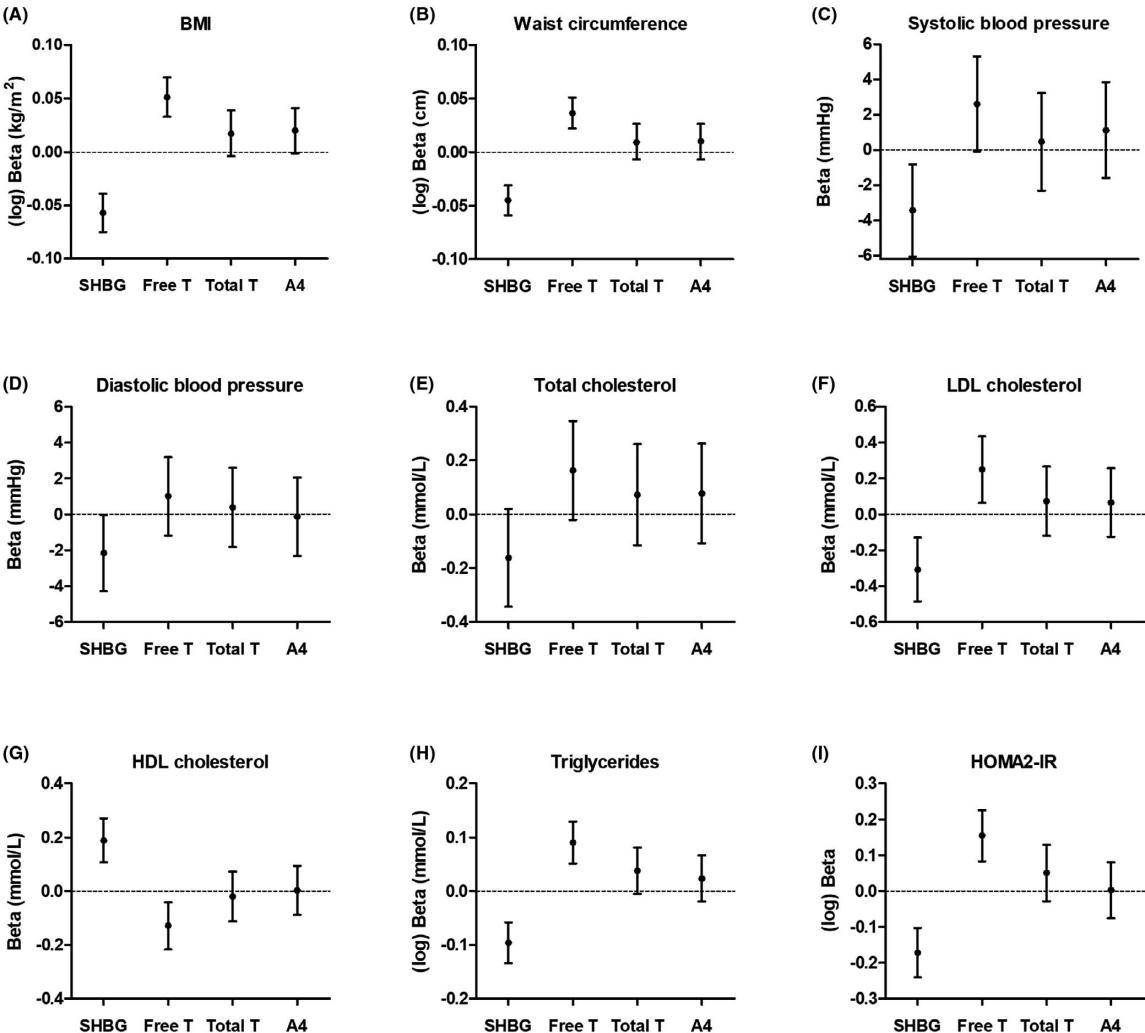
Associations of serum sex hormone‐binding globulin (SHBG), free testosterone (Free T), total testosterone (Total T) and androstenedione (A4) with metabolic features of polycystic ovary syndrome: BMI (n = 96) (A), waist circumference (n = 95) (B), systolic blood pressure (n = 96) (C), diastolic blood pressure (n = 96) (D), total cholesterol (n = 93) (E), LDL cholesterol (n = 93) (F), HDL cholesterol (n = 93) (G), triglycerides (n = 93) (H) and homeostatic model assessment of insulin resistance (HOMA2‐IR) (n = 92) (I). Analyses were conducted with *Z*‐scores to allow comparison. Regression coefficients should therefore be interpreted as the increase in the dependent variable per standard deviation increase in serum SHBG, free testosterone, total testosterone or androstenedione (after adjustment for fasting (yes/no), panel C‐I). See methods section

Figure 1 shows the associations of SHBG, androstenedione, total testosterone and free testosterone with nine metabolic features of PCOS. Serum SHBG and free testosterone, but not total testosterone or androstenedione, were associated with BMI (Figure 1A). Although similar patterns were observed for all other metabolic characteristics (Figure 1B‐I), statistical significance was reached for the relationship of both SHBG and free testosterone with waist circumference, LDL cholesterol, HDL cholesterol, serum triglycerides and HOMA2‐IR (Figure 1B, F‐I). Adjustment for age did not materially alter the strength of the statistically significant associations (Table 2). In contrast, additional adjustment for BMI reduced the strengths of all associations, whereas addition of HOMA2‐IR to the regression models did not have a substantial effect (Table 2). The strengths of associations did not substantially change when repeating the analyses in fasted individuals only (n = 89) (Figure S1 and Table S1).

**TABLE 2:** Associations of serum SHBG, free testosterone, total testosterone and androstenedione with metabolic and reproductive features of PCOS

Independent variables	SHBG beta (95% CI)	Free testosterone beta (95% CI)	Total testosterone beta (95% CI)	Androstenedione beta (95% CI)
Metabolic features				
(log) BMI				
Crude	**−0.06 (−0.08;−0.04)**	**0.05 (0.03;0.07)**	0.02 (0.00;0.04)	0.02 (0.00;0.04)
Age	**−0.06 (−0.07;−0.04)**	**0.05 (0.03;0.07)**	0.02 (−0.01;0.04)	0.02 (−0.01;0.04)
(log) Waist circumference				
Crude	**−0.05 (−0.06;−0.03)**	**0.04 (0.02;0.05)**	0.01 (−0.01;0.03)	0.01 (−0.01;0.03)
Age	**−0.05 (−0.06;−0.03)**	**0.04 (0.02;0.05)**	0.01 (−0.01;0.03)	0.01 (−0.01;0.03)
Systolic blood pressure				
Crude^a^	**−3.44 (−6.06;−0.82)**	2.62 (−0.08;5.31)	0.46 (−2.30;3.23)	1.12 (−1.60;3.84)
Age	**−4.00 (−6.65;−1.34)**	**3.29 (0.54;6.05)**	0.71 (−2.07;3.49)	1.86 (−0.99;4.71)
Age, BMI	−0.99 (−3.79;1.82)	0.10 (−2.75;2.94)	−0.49 (−2.97;1.98)	0.74 (−1.81;3.28)
Age, BMI, HOMA2‐IR	−0.93 (−3.73;1.88)	−0.30 (−3.23;2.63)	−0.90 (−3.46;1.65)	0.91 (−1.59;3.56)
Diastolic blood pressure				
Crude^a^	**−2.16 (−4.29;−0.04)**	1.00 (−1.19;3.20)	0.39 (−1.82;2.60)	−0.13 (−2.32;2.05)
Age	**−2.77 (−4.89;−0.66)**	1.70 (−0.51;3.92)	0.71 (−1.49;2.90)	0.63 (−1.63;2.90)
Age, BMI	−1.12 (−3.48;1.24)	−0.28 (−2.67;2.12)	0.00 (−2.08;2.09)	−0.05 (−2.20;2.10)
Age, BMI, HOMA2‐IR	−1.03 (−3.36;1.30)	0.86 (−3.30;1.57)	−0.53 (−2.66;1.61)	0.27 (−1.88;2.42)
LDL cholesterol				
Crude^a^	**−0.31 (−0.49;−0.13)**	**0.25 (0.06;0.44)**	0.07 (−0.13;0.27)	0.07 (−0.13;0.26)
Age	**−0.35 (−0.53;−0.17)**	**0.30 (0.11;0.49)**	0.09 (−0.10;0.28)	0.11 (−0.09;0.31)
Age, BMI	**−0.24 (−0.44;−0.03)**	0.17 (−0.04;0.38)	0.03 (−0.16;0.21)	0.06 (−0.16;0.25)
Age, BMI, HOMA2‐IR	**−0.24 (−0.44;−0.03)**	0.16 (−0.05;0.38)	0.01 (−0.18;0.21)	0.07 (−0.13;0.26)
HDL cholesterol				
Crude^a^	**0.19 (0.11;0.27)**	**−0.13 (−0.22;−0.04)**	−0.02 (−0.11;0.07)	0.00 (−0.09;0.09)
Age	**0.18 (0.09;0.26)**	**−0.11 (−0.20;−0.02)**	−0.01 (−0.10;0.08)	0.04 (−0.06;0.13)
Age, BMI	0.08 (−0.01;0.17)	0.00 (−0.09;0.09)	0.04 (−0.04;0.12)	0.08 (0.00;0.16)
Age, BMI, HOMA2‐IR	0.08 (−0.01;0.17)	0.02 (−0.07;0.12)	0.06 (−0.02;0.14)	0.07 (−0.01;0.15)
(log) Triglycerides				
Crude^a^	**−0.10 (−0.13;−0.06)**	**0.09 (0.05;0.13)**	0.04 (−0.01;0.08)	0.02 (−0.02;0.07)
Age	**−0.10 (−0.14;−0.06)**	**0.09 (0.05;0.13)**	0.04 (−0.01;0.08)	0.02 (−0.02;0.07)
Age, BMI	**−0.07 (−0.11;−0.02)**	**0.06 (0.01;0.10)**	0.02 (−0.02;0.06)	0.00 (−0.04;0.05)
Age, BMI, HOMA2‐IR	**−0.07 (−0.11;−0.02)**	**0.06 (0.01;0.10)**	0.02 (−0.03;0.06)	0.01 (−0.04;0.05)
(log) HOMA2‐IR				
Crude^a^	**−0.17 (−0.24;−0.10)**	**0.16 (0.08;0.23)**	0.05 (−0.03;0.13)	0.00 (−0.08;0.08)
Age	**−0.17 (−0.24;−0.10)**	**0.16 (0.08;0.23)**	0.05 (−0.03;0.13)	−0.01 (−0.09;0.07)
Age, BMI	−0.07 (−0.14;0.00)	0.05 (−0.02;0.12)	0.00 (−0.06;0.07)	−0.05 (−0.12;0.01)
Reproductive features				
Antral follicle count				
Crude	0.51 (−1.15;2.17)	1.38 (−0.26;3.02)	**1.99 (0.38;3.59)**	1.12 (−0.53;2.78)
Age	0.83 (−0.85;2.51)	1.10 (−0.60;2.81)	**1.82 (0.19;3.45)**	0.81 (−0.94;2.56)
Age, BMI	0.78 (−1.18;2.74)	1.75 (−0.19;3.68)	**1.97 (0.32;3.62)**	0.93 (−0.85;2.70)
Age, BMI, HOMA2‐IR^b^	0.86 (−1.07;2.79)	1.28 (−0.72;3.27)	1.58 (−0.15;3.31)	1.25 (−0.52;3.01)
(log) AMH				
Crude	**0.07 (0.01;0.12)**	−0.01 (−0.07;0.04)	0.05 (0.00;0.11)	**0.06 (0.00;0.11)**
Age	**0.08 (0.02;0.13)**	−0.02 (−0.08;0.04)	0.05 (−0.01;0.10)	0.05 (−0.01;0.11)
Age, BMI	0.04 (−0.03;0.10)	0.03 (−0.03;0.09)	**0.07 (0.01;0.12)**	**0.07 (0.02;0.13)**
Age, BMI, HOMA2‐IR^b^	0.04 (−0.03;0.10)	0.03 (−0.03;0.10)	**0.07 (0.01;0.13)**	**0.07 (0.02;0.13)**

Analyses were conducted with Z‐scores. Beta coefficients should therefore be interpreted as per standard deviation increase in serum SHBG, free testosterone, total testosterone or androstenedione. See methods section.

Bold values indicate statistical significance (*p* < .05).

Abbreviations: AMH, anti‐Müllerian hormone; BMI, body mass index; HDL, high‐density lipoprotein; HOMA2‐IR, homeostatic model assessment for insulin resistance; LDL, low‐density lipoprotein; SHBG, sex hormone‐binding globulin.

^a^ Adjusted for fasting (yes/no) in all models.

^b^ Model additionally adjusted for fasting (yes/no).

### 3.3 Associations of serum SHBG, androstenedione, total testosterone and free testosterone with reproductive features of PCOS

**FIGURE 2:**
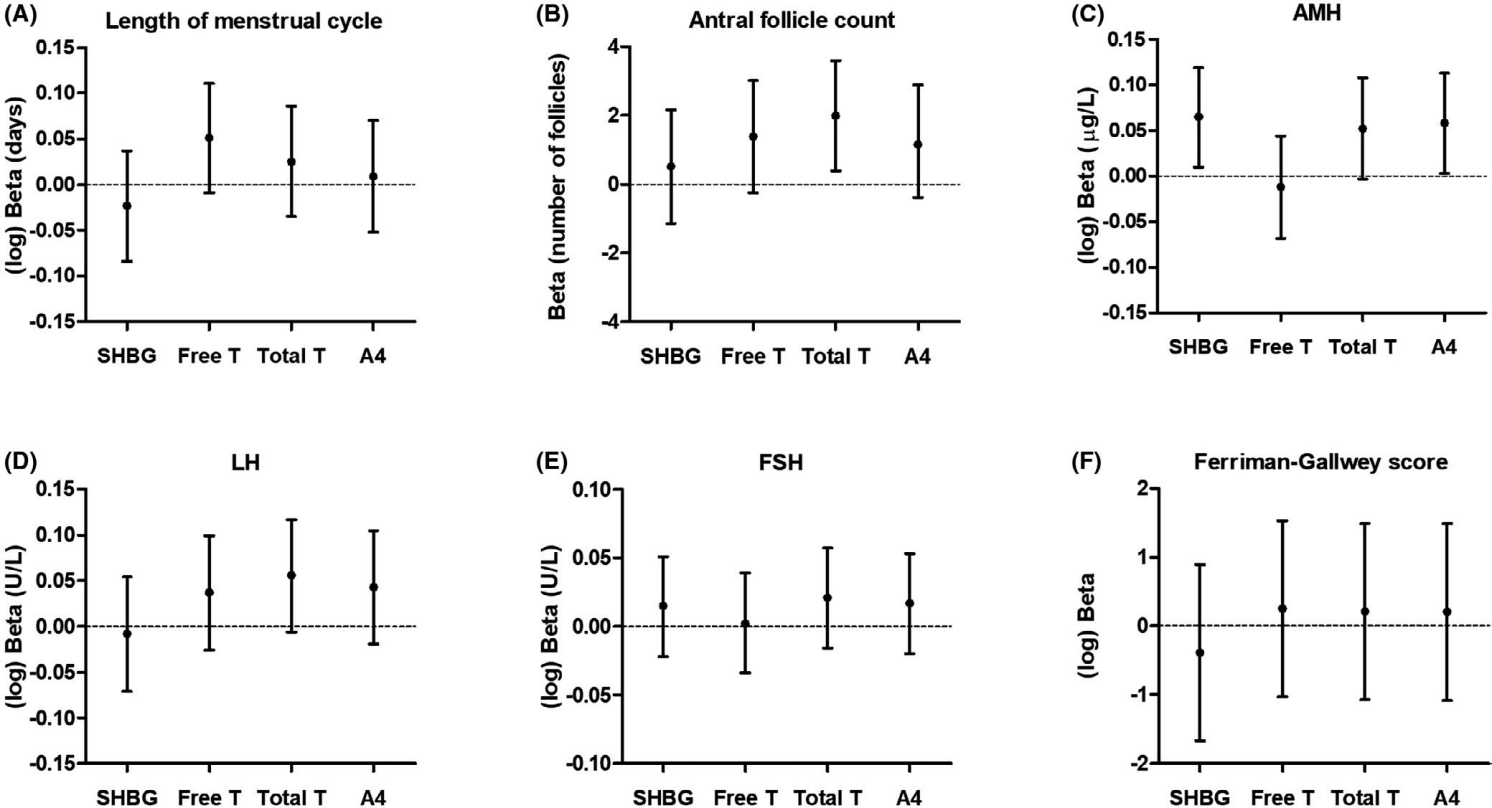
Associations of serum sex hormone‐binding globulin (SHBG), free testosterone (Free T), total testosterone (Total T) and androstenedione (A4) with reproductive features of polycystic ovary syndrome: length of menstrual cycle (n = 89) (A), antral follicle count (n = 92) (B), anti‐Müllerian hormone (AMH) (n = 92) (C), luteinizing hormone (LH) (n = 95) (D), follicle‐stimulating hormone (FSH) (n = 96) (E) and Ferriman‐Gallwey score (n = 96) (F). Analyses were conducted with *Z*‐scores to allow comparison. Regression coefficients should, therefore, be interpreted as the increase in the dependent variable per standard deviation increase in serum SHBG, free testosterone, total testosterone or androstenedione. See methods section

Figure 2 shows the relationships of SHBG, androstenedione, total testosterone and free testosterone with six reproductive features of PCOS. None of these were associated with menstrual cycle length (Figure 2A). Total testosterone, but not SHBG, was statistically significantly associated with antral follicle count (Figure 2B), which was not affected by adjustment for age and BMI (Table 2). The strength of association was reduced and no longer statistically significant after further adjustment for HOMA2‐IR (Table 2). Androstenedione and SHBG were significantly associated with serum AMH (Figure 2C). The significant association between SHBG and serum AMH was lost after adjustment for age and BMI (Table 2). Total testosterone was significantly associated with serum AMH after adjustment for age and BMI (Table 2). No significant associations were observed for serum LH and FSH (Figure 2D,E, respectively). Finally, although the direction of the associations of serum SHBG, androstenedione, total testosterone and free testosterone with the Ferriman‐Gallwey score were as anticipated, that is inverse for SHBG and positive for androstenedione, total testosterone, and free testosterone, none of these associations were statistically significant (Figure 2F). The strengths of associations did not substantially change when repeating the analyses in fasted individuals only (n = 89) (Figure S2 and Table S1).

## 4 DISCUSSION

The aim of this study was to examine the associations of SHBG, androstenedione, total testosterone and free testosterone with the individual metabolic and reproductive features of PCOS. Serum SHBG and free testosterone, but not total testosterone or androstenedione, were significantly associated with BMI, waist circumference, serum triglycerides, HDL cholesterol, LDL cholesterol and HOMA2‐IR. In contrast, in the adjusted models total testosterone was significantly associated with antral follicle count and serum AMH, while androstenedione was significantly associated with serum AMH. Adjustment for BMI substantially reduced the strength of association of free testosterone and SHBG with the metabolic features of PCOS, but hardly affected the associations of total testosterone or androstenedione with the reproductive features of PCOS.

The observed patterns of associations, that is SHBG mainly associates with metabolic features whereas total testosterone and androstenedione associate with reproductive abnormalities of PCOS, are in line with previous observational studies in PCOS.^11^, ^12^, ^13^, ^14^ A recent Mendelian randomization study showed that genetically predicted SHBG, total testosterone and free testosterone levels were associated with PCOS risk,^5^ which is not surprising given the adoption of hyperandrogenism as a diagnostic criterion of PCOS.^15^ However, PCOS is a complex disorder, which comprises several metabolic and ovarian sub‐phenotypes.^2^, ^25^ The patterns of associations seen in this study, support that different features of PCOS could have a unique aetiology with diverse, though potentially intertwining, pathophysiological pathways.

Experimental studies have shown that hepatic de novo lipogenesis, which is increased in obesity and insulin resistance,^26^ impairs SHBG synthesis in the liver.^27^ We recently demonstrated that de novo lipogenesis, assessed by stable isotopes, is inversely associated with serum SHBG levels in women.^28^ Hepatic de novo lipogenesis has also been associated with a disadvantageous lipid profile.^29^ The reduction in the strength of the association between SHBG and serum lipids after adjustment for BMI in the current study is in line with these previous observations and suggests that BMI is an important driver of the metabolic features and low serum SHBG levels that characterize PCOS.^30^ Indeed, a recent bidirectional Mendelian randomization study suggested that BMI is causal in the development of PCOS, but not vice versa.^31^ Furthermore, other Mendelian randomization studies have demonstrated that free testosterone, SHBG and total testosterone do not appear to exert a causal effect on BMI, serum lipids and blood pressure.^5^, ^32^, ^33^ In contrast, Mendelian randomization studies have suggested that SHBG is actively involved in the pathogenesis of type 2 diabetes, either directly or via free testosterone.^5^, ^34^, ^35^ This is supported by experimental studies in humanized transgenic *SHBG* mice that were fed a high‐fat diet and demonstrated an improved glucose homeostasis compared to wild‐type mice.^36^

The pathophysiology of the reproductive features of PCOS is not as well understood. Elevated AMH levels—a result of impaired follicle development and an increased number of antral follicles—have been linked to ovarian androgen hypersecretion by inhibition of the aromatase‐induced conversion of androgens to oestrogens and stimulation of GnRH‐dependent LH secretion.^2^, ^10^, ^37^, ^38^ Simultaneously, hyperandrogenism may increase AMH levels through its proposed disruptive effects on follicular development.^2^ Insulin resistance has also been indicated as an important contributor to ovarian androgen secretion,^39^, ^40^ yet we did not find a significant association between total testosterone or androstenedione with HOMA2‐IR. The mechanism by which insulin resistance influences reproductive features of PCOS therefore deserves further investigation.

In the current study, there was no association between any of the androgen markers and the Ferriman‐Gallwey score. Although this may be the result of insufficient statistical power, it also corroborates a recent meta‐regression analysis in 6593 women with PCOS demonstrating that free testosterone levels were not associated with clinical hyperandrogenism.^41^ Hirsutism is a phenotypic expression of several factors, including the androgen concentration, androgen receptor activity and 5‐α reductase activity at the pilosebaceous units.^41^ Furthermore, the Ferriman‐Gallwey score is a subjective measure with significant interobserver variability.^42^ Both facets may contribute to the lack of an association between free testosterone levels and clinical hyperandrogenism.

The differential patterns of associations as observed in the present study indicate that PCOS constitutes a heterogeneous phenotype. The recent ESHRE guideline relies primarily on markers of free testosterone as a diagnostic tool for biochemical hyperandrogenism.^18^ This allows the diagnosis of a broad range of PCOS phenotypes, as is also supported by the current findings. However, SHBG and total testosterone levels may guide clinicians in determining the primary contributing pathway (ie metabolic or reproductive) in individual patients. The extent to which either pathway is involved may vary greatly between individuals. Clinical follow‐up and management of patients with PCOS might benefit from a more targeted approach, based on the primary underlying pathway, which warrants further study.

This study has several strengths and limitations. The PCOS cohort examined in this study has been systematically screened in a hospital setting, allowing us to gather information on a wide range of PCOS features. This setting, that is an outpatient clinic for menstrual cycle disorders, could have resulted in a selection of a particular subtype of PCOS. The interpretation of the results provides insight into the underlying pathways using observational data, but warrant further studies to unravel the exact role of free testosterone in the pathogenesis of the individual PCOS features. Finally, the relatively small sample size could have resulted in a lack of statistical power and, hence, type 2 errors. Indeed, several associations approached, but did not reach, statistical significance (Figures 1 and 2).

In conclusion, the current observational study shows differential associations of SHBG, androstenedione, total testosterone and free testosterone levels with metabolic and reproductive features of PCOS. These differential associations highlight the heterogeneous nature of PCOS and suggest that the underlying pathways contributing to the features of PCOS are diverse. The combination of SHBG, total testosterone and androstenedione levels may provide information on the primary underlying pathophysiological pathway in women with PCOS.

## CONFLICT OF INTEREST

The authors declare that there is no conflict of interest.

## AUTHOR CONTRIBUTIONS

P.I.H.G. Simons involved in investigation, formal analysis and writing‐original draft preparation. O. Valkenburg involved in investigation, data curation, and writing‐review and editing. J.A.P. Bons involved in resources, and writing‐review and editing. C.D.A. Stehouwer involved in writing‐review and editing. M.C.G.J. Brouwers involved in conceptualization, supervision, funding acquisition, and writing‐review and editing.

## Supporting information

Supplementary Material

## Data Availability

The data that support the findings of this study are available from the corresponding author upon reasonable request.
