# Supplementary material for: The relationships of sex hormone‐binding globulin, total testosterone, androstenedione and free testosterone with metabolic and reproductive features of polycystic ovary syndrome
Source: Endocrinol Diabetes Metab. 2021 May 24;4(3):e00267. doi: 10.1002/edm2.267 (PMC8279613; doi:10.1002/edm2.267)
Supplement: Supplementary file 1 — Supplementary Material [file EDM2-4-e00267-s001.docx]

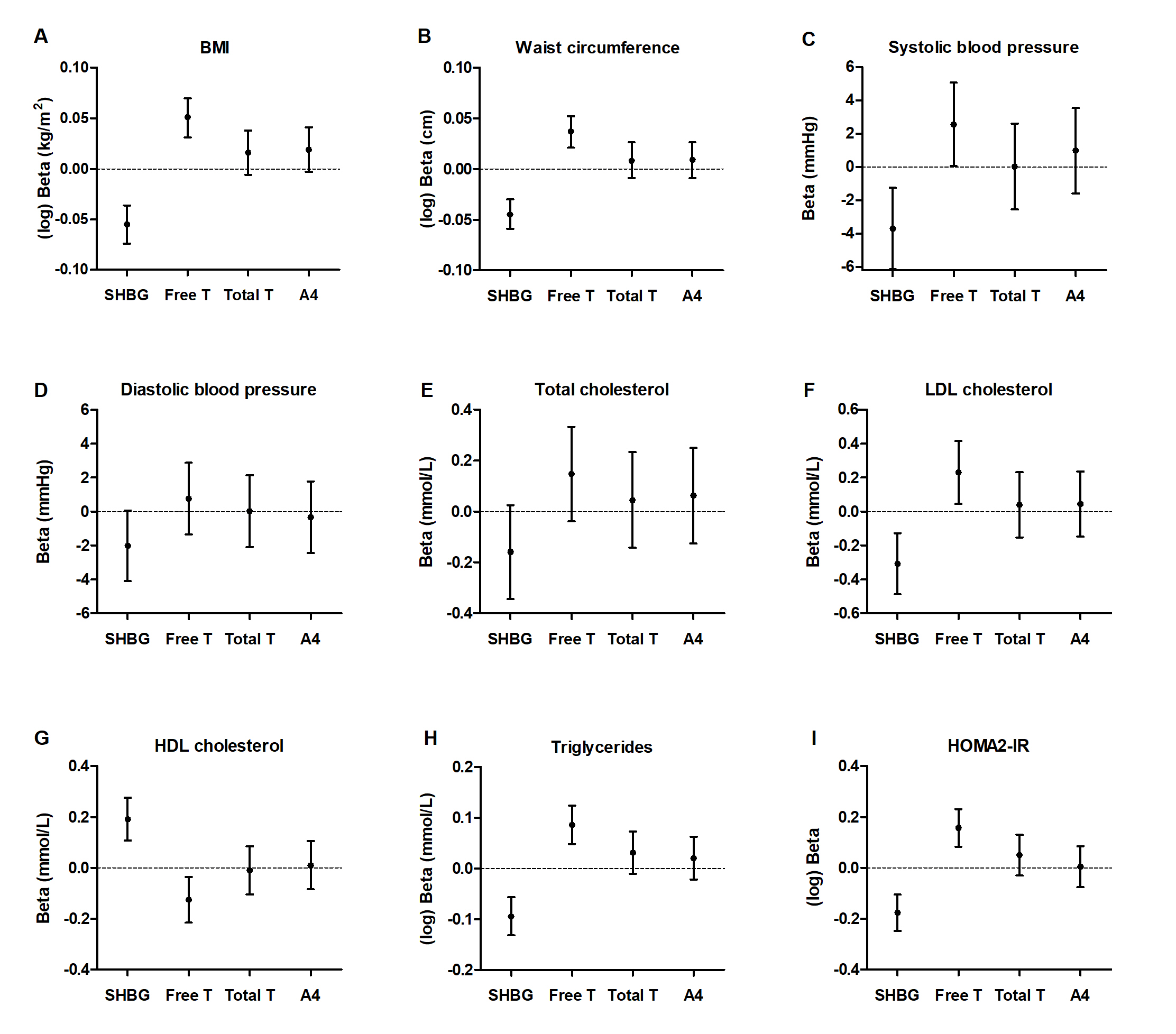


**Supplementary Figure 1.** Associations of serum sex hormone-binding globulin (SHBG), free testosterone (Free T), total testosterone (Total T), and androstenedione (A4) with metabolic features of polycystic ovary syndrome in fasted individuals only: BMI (n = 89) (A) waist circumference (n = 88) (B) systolic blood pressure (n = 89) (C) diastolic blood pressure (n = 89) (D) total cholesterol (n = 88) (E) LDL cholesterol (n = 88) (F) HDL cholesterol (n = 88) (G) triglycerides (n = 88) (H) and homeostatic model assessment of insulin resistance (HOMA2-IR) (n = 87) (I).

Analyses were conducted with Z-scores to allow comparison. Regression coefficients should therefore be interpreted as the increase in the dependent variable per standard deviation increase in serum SHBG, free testosterone, total testosterone or androstenedione. See methods section.


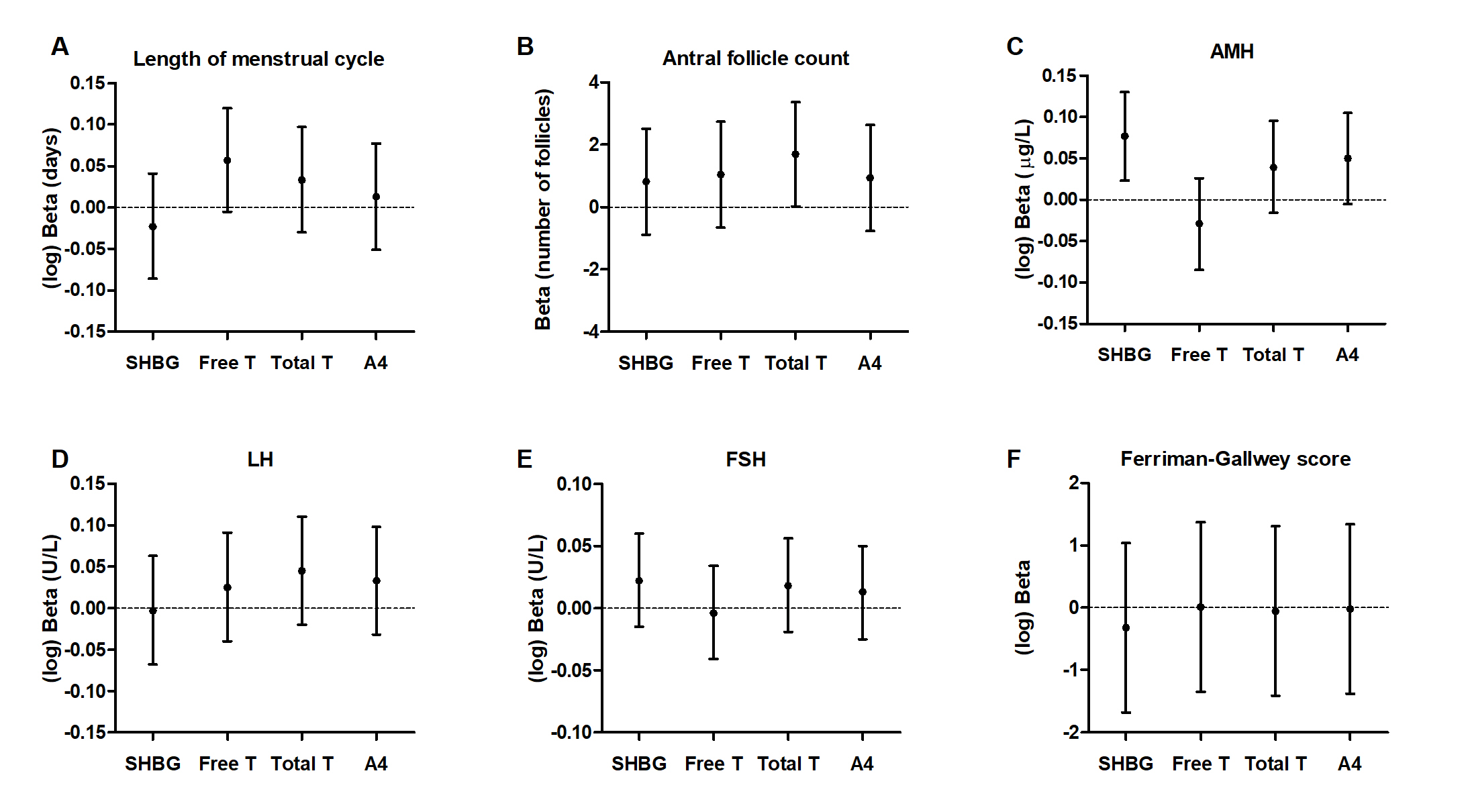


**Supplementary Figure 2.** Associations of serum sex hormone-binding globulin (SHBG), free testosterone (Free T), total testosterone (Total T), and androstenedione (A4) with reproductive features of polycystic ovary syndrome in fasted individuals only: length of menstrual cycle (n = 83) (A) antral follicle count (n = 85) (B) anti-Mullerian hormone (AMH) (n = 86) (C) luteinizing hormone (LH) (n = 89) (D) follicle stimulating hormone (FSH) (n = 89) (E) and Ferriman-Gallwey score (n = 89) (F).

Analyses were conducted with Z-scores to allow comparison. Regression coefficients should, therefore, be interpreted as the increase in the dependent variable per standard deviation increase in serum SHBG, free testosterone, total testosterone or androstenedione. See methods section.

**Supplementary Table 1. Association of serum SHBG, free testosterone, total testosterone and androstenedione with metabolic and reproductive features of PCOS in fasted individuals only.**

| **Independent variables** | **SHBG**  **beta (95% CI)** | **Free testosterone**  **beta (95% CI)** | **Total testosterone**  **beta (95% CI)** | **Androstenedione**  **beta (95% CI)** |
| --- | --- | --- | --- | --- |
| **Metabolic features** |  |  |  |  |
| *(log) BMI* |  |  |  |  |
| Crude | **-0.06 (-0.07;-0.04)** | **0.05 (0.03;0.07)** | 0.02 (-0.01;0.04) | 0.02 (0.00;0.04) |
| Age | **-0.05 (-0.07;-0.04)** | **0.05 (0.03;0.07)** | 0.01 (-0.01;0.04) | 0.02 (-0.01;0.04) |
| *(log) Waist circumference* |  |  |  |  |
| Crude | **-0.05 (-0.06;-0.03)** | **0.04 (0.02;0.05)** | 0.01 (-0.01;0.03) | 0.01 (-0.01;0.03) |
| Age | **-0.05 (-0.06;-0.03)** | **0.04 (0.02;0.05)** | 0.01 (-0.01;0.03) | 0.01 (-0.01;0.03) |
| *Systolic blood pressure* |  |  |  |  |
| Crude | **-3.69 (-6.45;-1.24)** | **2.56 (0.04;5.08)** | 0.03 (-2.55;2.60) | 0.98 (-1.59;3.55) |
| Age | **-4.00 (-6.49;-1.50)** | **2.91 (0.32;5.50)** | 0.12 (-2.49;2.72) | 1.33 (-1.37;4.02) |
| Age, BMI | -1.67 (-3.76;1.42) | -0.28 (-2.90;2.35) | -1.02 (-3.28;1.25) | 0.13 (-2.24;2.50) |
| Age, BMI, HOMA2-IR | -1.12 (-3.72;1.48) | -0.63 (-3.33;2.07) | -1.43 (-3.77;0.91) | 0.35 (-2.05;2.76) |
| *Diastolic blood pressure* |  |  |  |  |
| Crude | -2.02 (-4.09;0.05) | 0.76 (-1.35;2.87) | 0.03 (-2.08;2.15) | -0.33 (-2.45;1.78) |
| Age | **-2.52 (-4.57;-0.47)** | 1.33 (-0.79;3.46) | 0.27 (-1.82;2.37) | 0.30 (-1.88;2.48) |
| Age, BMI | -1.21 (-3.50;1.09) | -0.36 (-2.69;1.97) | -0.31 (-2.32;1.71) | -0.33 (-2.43;1.77) |
| Age, BMI, HOMA2-IR | -1.11 (-3.36;1.14) | -1.01 (-3.35;1.33) | -0.95 (-3.00;1.09) | 0.07 (-2.02;2.16) |
| *LDL cholesterol* |  |  |  |  |
| Crude | **-0.31 (-0.49;-0.13)** | **0.23 (0.05;0.42)** | 0.04 (-0.15;0.23) | 0.05 (-0.15;0.24) |
| Age | **-0.34 (-0.52;-0.16)** | **0.27 (0.08;0.46)** | 0.05 (-0.14;0.28) | 0.08 (-0.12;0.28) |
| Age, BMI | **-0.23 (-0.43;-0.03)** | 0.14 (-0.08;0.35) | -0.01 (-0.19;0.18) | 0.01 (-0.18;0.21) |
| Age, BMI, HOMA2-IR | **-0.23 (-0.43;-0.02)** | 0.13 (-0.09;0.35) | -0.02 (-0.22;0.17) | 0.02 (-0.17;0.22) |
| *HDL cholesterol* |  |  |  |  |
| Crude | **0.19 (0.11;0.28)** | **-0.13 (-0.22;-0.04)** | -0.01 (-0.10;0.08) | 0.01 (-0.08;0.11) |
| Age | **0.18 (0.09;0.26)** | **-0.11 (-0.20;-0.01)** | 0.00 (-0.09;0.10) | 0.05 (-0.05;0.14) |
| Age, BMI | 0.08 (-0.01;0.17) | 0.02 (-0.08;0.11) | 0.05 (-0.03;0.12) | **0.09 (0.01;0.17)** |
| Age, BMI, HOMA2-IR | 0.08 (-0.01;0.17) | 0.03 (-0.06;0.13) | 0.06 (-0.02;0.14) | **0.09 (0.06;0.17)** |
| *(log) Triglycerides* |  |  |  |  |
| Crude | **-0.10 (-0.13;-0.06)** | **0.09 (0.05;0.12)** | 0.03 (-0.01;0.07) | 0.02 (-0.02;0.06) |
| Age | **-0.09 (-0.13;-0.06)** | **0.09 (0.05;0.13)** | 0.03 (-0.01;0.07) | 0.02 (-0.03;0.06) |
| Age, BMI | **-0.07 (-0.11;-0.02)** | **0.05 (0.01;0.10)** | 0.01 (-0.02;0.05) | 0.00 (-0.04;0.04) |
| Age, BMI, HOMA2-IR | **-0.06 (-0.11;-0.02)** | **0.05 (0.01;0.10)** | 0.01 (-0.03;0.05) | 0.01 (-0.04;0.04) |
| *(log) HOMA2-IR* |  |  |  |  |
| Crude | **-0.18 (-0.25;-0.11)** | **0.16 (0.08;0.23)** | 0.05 (-0.03;0.13) | 0.01 (-0.08;0.09) |
| Age | **-0.17 (-0.25;-0.10)** | **0.16 (0.08;0.23)** | 0.05 (-0.04;0.13) | -0.01 (-0.10;0.08) |
| Age, BMI | -0.07 (-0.14;0.00) | 0.04 (-0.03;0.12) | 0.00 (-0.06;0.07) | -0.06 (-0.12;0.01) |
|  |  |  |  |  |
| **Reproductive features** |  |  |  |  |
| *Antral follicle count* |  |  |  |  |
| Crude | 0.81 (-0.89;2.52) | 1.04 (-0.66;2.74) | **1.70 (0.22;3.37)** | 0.93 (-0.78;2.63) |
| Age | 1.07 (-0.65;2.80) | 0.81 (-0.94;2.56) | 1.59 (-0.10;3.27) | 0.65 (-1.13;2.43) |
| Age, BMI | 1.11 (-0.88;3.10) | 1.37 (-0.63;3.37) | **1.72 (0.01;3.43)** | 0.76 (-1.06;2.58) |
| Age, BMI, HOMA2-IR | 1.19 (-0.77;3.14) | 0.95 (-1.09;3.00) | 1.35 (-0.41;3.11) | 1.12 (-0.69;2.92) |
| *(log) AMH* |  |  |  |  |
| Crude | **0.08 (0.02;0.13)** | -0.03 (-0.09;0.03) | 0.04 (-0.02;0.10) | 0.05 (-0.01;0.11) |
| Age | **0.08 (0.03;0.14)** | -0.04 (-0.09;0.02) | 0.04 (-0.02;0.09) | 0.05 (-0.01;0.11) |
| Age, BMI | 0.05 (-0.01;0.11) | 0.01 (-0.05;0.07) | **0.06 (0.00;0.11)** | **0.07 (0.01;0.12)** |
| Age, BMI, HOMA2-IR | 0.05 (-0.01;0.11) | 0.02 (-0.05;0.08) | **0.06 (0.01;0.13)** | **0.07 (0.01;0.12)** |

Analyses were conducted with Z-scores. Beta coefficients should therefore be interpreted as per standard deviation increase in serum SHBG, free testosterone, total testosterone or androstenedione. See methods section.

Bold values indicate statistical significance (p < 0.05).

Abbreviations: AMH: anti-Mullerian hormone; BMI: body mass index; HOMA2-IR: homeostatic model assessment for insulin resistance; HDL: high-density lipoprotein; LDL: low-density lipoprotein; AMH: anti-Mullerian hormone; SHBG: sex hormone-binding globulin.
